# Short-term and long-term outcomes of intrathoracic vacuum therapy of empyema in debilitated patients

**DOI:** 10.1186/s13019-016-0543-7

**Published:** 2016-10-21

**Authors:** Zsolt Sziklavari, Michael Ried, Florian Zeman, Christian Grosser, Tamas Szöke, Reiner Neu, Rudolf Schemm, Hans-Stefan Hofmann

**Affiliations:** 1 Department of Thoracic Surgery, Krankenhaus Barmherzige Brüder Regensburg, Prüfeningerstraße 86, 93049 Regensburg, Germany; 2 Department of Thoracic Surgery, University Medical Center Regensburg, Franz-Josef-Strauss-Allee 11, 93053 Regensburg, Germany; 3 Center for Clinical Studies, University Medical Center Regensburg, Franz-Josef-Strauss-Allee 11, 93053 Regensburg, Germany

**Keywords:** Negative pressure wound therapy, VAC, Vacuum-assisted closure, Intrapleural, Intrathoracic, Empyema

## Abstract

**Background:**

This retrospective study analyzed the effectiveness of intrathoracic negative pressure therapy for debilitated patients with empyema and compared the short-term and long-term outcomes of three different intrapleural vacuum-assisted closure (VAC) techniques.

**Methods:**

We investigated 43 consecutive (pre)septic patients with poor general condition (Karnofsky index ≤ 50 %) and multimorbidity (≥ 3 organ diseases) or immunosuppression, who had been treated for primary, postoperative, or recurrent pleural empyema with VAC in combination with open window thoracostomy (OWT-VAC) with minimally invasive technique (Mini-VAC), and instillation (Mini-VAC-Instill).

**Results:**

The overall duration of intrathoracic vacuum therapy was 14 days (5–48 days). Vacuum duration in the Mini-VAC and Mini-VAC-Instill groups (12.4 ± 5.7 and 10.4 ± 5.4 days) was significantly shorter (*p* = 0.001) than in the group treated with open window thoracostomy (OWT)-VAC (20.3 ± 9.4 days). No major complication was related to intrathoracic VAC therapy. Chest wall closure rates were significantly higher in the Mini-VAC and Mini-VAC-Instill groups than in the OWT-VAC group (*p* = 0.034 and *p* = 0.026). Overall, the mean postoperative length of stay in hospital (LOS) was 21 days (median 18, 6–51 days). LOS was significantly shorter (*p* = 0.027) in the Mini-VAC-Instill group (15.1 ± 4.8) than in the other two groups (23.8 ± 12.3 and 22.7 ± 1.5). Overall, the 30-day and 60-day mortality rates were 4.7 % (2/43) and 9.3 % (4/43), and none of the deaths was related to infection.

**Conclusions:**

For debilitated patients, immediate minimally invasive intrathoracic vacuum therapy is a safe and viable alternative to OWT. Mini-VAC-Instill may have the fastest clearance and healing rates of empyema.

## Background

**Fig. 1:**
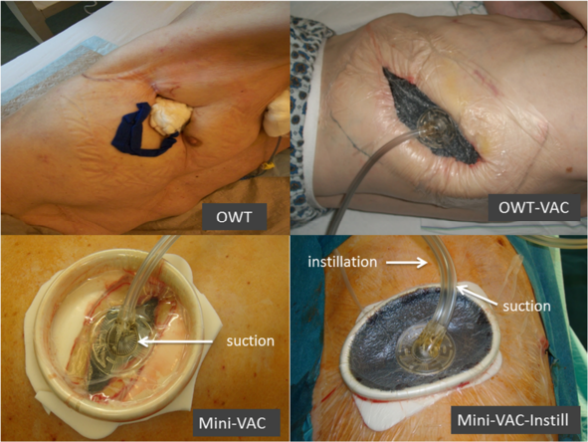
OWT, OWT-VAC, Mini-VAC, Mini-VAC-Instill

Reports on pleural diseases and therapeutic modalities in the pleural cavity go back for centuries, and particularly thoracic empyema has been a source of fascination for physicians from different cultures. Nowadays, treatment of pleural empyema generally depends on the estimated stage of disease progression [1]. In critically ill patients or in the case of recurrent empyema, surgical treatment still represents a big challenge. In such cases, open window thoracostomy (OWT) allows rapid evacuation of pus, extensive debridement, and decortication. Fenestration of the chest wall is an ideal method for accelerating drainage in patients with bronchial stump insufficiency, bronchopleural fistula, or pleural empyema. Nevertheless, OWT has several disadvantages; for instance, this method requires division of the chest wall muscles and resection of several ribs, resulting in a partial thorax defect (Fig. 1a). OWT also necessitates obliteration of the pleural space, which, in turn, requires the often painful and time-consuming procedure of packing on a daily basis. In addition, the chest wall should be closed; however, chest wall closure after OWT is not always possible [2].

In contrast, previous studies [3] have shown that accelerated OWT empyema drainage and resolution may be achieved with intrathoracic vacuum-assisted closure (OWT-VAC and also NPWT: negative pressure wound therapy). Intrathoracic negative pressure therapy can be minimally invasive without altering the integrity of the chest wall (Mini-VAC) [4]. Various modifications have been reported for treatment optimization, such as vacuum-assisted closure with instillation (Mini-VAC-Instill) [5]. Recent studies have shown that Mini-VAC and Mini-VAC-Instill may produce equivalent resolution rates and better OWT closure rates than open approaches [6].

Here, we report our biggest cohort to date of 43 debilitated or septic patients with pleural empyema due to various etiologies. This retrospective study investigated the effectiveness of intrathoracic negative pressure therapy for debilitated patients with empyema and compared the short-term and long-term outcomes of three different intrapleural vacuum-assisted closure (VAC) techniques.

## Methods

### Patients

Between September 2009 and December 2014, 379 patients had been treated for primary and postoperative pleural empyema at the Center of Thoracic Surgery in Regensburg, Germany (Krankenhaus Barmherzige Brüder Regensburg, University Medical Center Regensburg). In this retrospective study, we investigated 43 of 379 (11.34 %) consecutive (pre)septic patients with poor general condition (Karnofsky index ≤ 50 %) and multimorbidity (≥ 3 organ diseases) or immunosuppression, who had been treated for primary, postoperative, or recurrent pleural empyema with intrapleural VAC therapy. We reviewed the medical records of all patients with intrapleural VAC; no patient was excluded. The study was reviewed and approved by the Ethics Committee of the University of Regensburg.

### Clinical features

Parameters as well as demographic and clinical characteristics are summarized in Table 1. Seventeen (40 %) of 43 patients had presented with parapneumonic or postpneumonic empyema. Twenty-six (60 %) of 43 patients had postoperative pleural empyema, of whom six patients presented with postpneumonectomy (three of these six patients had undergone completion pneumectomy due to postlobectomy empyema), five with postlobectomy empyema, and five with bronchopleural fistula (BPF). Two patients had esophageal-pulmonary fistula. The remaining 8 patients had developed empyema after other thoracic procedures. Indication for surgery was thoracic empyema at American Thoracic Society (ATS) stage II (*n* = 37) and stage III (*n* = 6) and pre-septic or septic condition. Four patients had developed septic shock.

**Table 1:** Patient demographics with non-parametric methods for testing whether clinical features and samples of OWT-VAC, Mini-VAC, and Mini-VAC-Instill originate from the same distribution

Baseline characteristic	Total (*n* = 43)	OWT-VAC (*n* = 20)	Mini-VAC (*n* = 8)	Mini-VAC-Instill (*n* = 15)	*p*-value
Age [years], mean (range)	64 (25–91)	65 (39–85)	57 (43–68)	67 (25–91)	0.243^a^
Sex, *n* (%)					0.237^c^
Women	4/43 (9 %)	1/20 (5 %)	0/8 (0 %)	3/15 (20 %)	
Men	39/43 (91 %)	19/20 (95 %)	8/8 (100 %)	12/15 (80 %)	
Multimorbidity	32/43 (74 %)	15/20 (75 %)	7/8 (88 %)	10/15 (67 %)	0.534^c^
Empyema side, *n* (%)					0.039^c^
Right	27/43 (63 %)	11/20 (55 %)	3/8 (38 %)	13/15 (87 %)	
Left	16/43 (37 %)	9/20 (45 %)	5/8 (62 %)	2/15 (13 %)	
Immunosuppression, *n* (%)	19/43 (44 %)	8/20 (40 %)	4/8 (50 %)	7/15 (47 %)	0.851^c^
Previous antibiotics, *n* (%)	28/43 (65 %)	11/20 (55 %)	7/8 (88 %)	10/15 (67 %)	0.285^c^
Malignancy	19/43 (44 %)	11/20 (55 %)	3/8 (38 %)	5/15 (33 %)	0.443^c^
Empyema genesis, *n* (%)					0.057^c^
Primary	17/43 (40 %)	4/20 (20 %)	5/8 (62 %)	8/15 (53 %)	
Secondary-postoperative	26/43 (60 %)	16/20 (80 %)	3/8 (38 %)	7/15 (47 %)	
ATS empyema stage, *n* (%)					0.464^c^
Stage II	37/43 (86 %)	17/20 (85 %)	8/8 (100 %)	12/15 (80 %)	
Stage III	6/43 (14 %)	3/20 (15 %)	0/8 (0 %)	3/15 (20 %)	
Recurrent empyema with previous treatment, *n* (%)	28/43 (65 %)	13/20 (65 %)	7/8 (88 %)	8/15 (53 %)	0.285^c^

^a^ANOVA, ^c^Chi: Exact Pearson’s chi-squared Test

The patients were grouped according to the VAC treatment modality received (OWT-VAC vs. Mini-VAC vs. Mini-VAC-Instill). Grouping was done by chronologic selection: OWT-VAC (*n* = 20) procedures were carried out between 2009 and 2011, Mini-VAC (*n* = 8) between 2011 and 2013, and Mini-VAC-Instill (*n* = 15) from 2014 onwards. The three groups did not substantially differ with regard to clinical features (Table 1). Some evaluations were also conducted according to the genesis of pleural empyema (primary vs. postoperative).

### Operative technique

**OWT-VAC group** (2009 to 2011, *n* = 20). Surgery consisting of OWT and VAC (Fig. 1b) under general anesthesia included CT-guided partial resection of 2 to 4 ribs (marsupialization), pus evacuation, debridement, local decortication, and flushing of the cavity with ringer solution and 10 % Betaisodona (Povidon-Iod, Mundipharma, Limburg, Germany) solution. Suturing the skin flaps on the margins of the OWT constituted thoracostoma. VAC sponges (black GranuFoam, Standard Dressings Kits, KCI Medical, Wiesbaden, Germany) were inserted into the residual pleural cavity through the thoracostoma to fill the entire pleural space. Suction was normally set to −100 mmHg from the start (maximum suction −125 mmHg) and to −75 mmHg after pneumonectomy. Sponges were changed twice a week under intravenous propofol anesthesia with short laryngeal mask airway ventilation. After the inpatient stay, OWT was either treated with continuing VAC therapy on an outpatient basis or with conventional wound care in an outpatient clinic. The chest wall was closed after an average of 3 months. Persistent BPF was treated with additional muscle flap transposition (*n* = 2) or pericardial flap (*n* = 3).

**Mini-VAC group** (2011 to 2013, *n* = 8). Patients of this group underwent mini-thoracotomy (thoracotomy with a 5–6 cm incision without insertion of a rib spreader) followed by the positioning of an ALEXIS® (Applied Medical, Rancho Santa Margarita, CA, USA) retractor (Fig. 1c). After debridement, local decortication, and flushing of the pleural cavity, VAC sponges were inserted through the tissue retractor to fill the entire pleural space. The level of suction was set to −125 mmHg from the start. Sponges were changed twice a week under short general anesthesia. The residual space was filled with an aminoglycoside antibiotic gentamicin sponge (Genta-Coll® resorb, Resorba, Nürnberg, Germany).

**Mini-VAC-Instill group** (2013 to 2014, *n* = 15). This updated procedure is a combination of Mini-VAC and intrapleural instillation of antiseptic fluid [5]. After debridement, the cavity was flushed with polyhexanide solution (LAVANID® 0.02 %, Serag-Wiessner KG, Naila, Germany). Special sponges (Instill Dressings, KCI Medical, Wiesbaden, Germany) were introduced via the soft tissue retractor. Here, the VAC pad had two tubes: one for suction and one for fluid instillation (Fig. 1d). The amount of instilled antiseptic volume was determined by the volume of the pleural wound. The level of suction was set to −75 mmHg from the start with a maximum of −125 mmHg. The system had a program of automated fluid instillation and vacuum cycles of 20 min six times a day. The Mini-VAC-(Instill) system was changed every Tuesday and Friday under intravenous propofol anesthesia with short laryngeal mask airway ventilation. Only minor debridement was required at each sponge change. In all patients, the remaining pleural space after Mini-VAC-Instill therapy was very small and only filled with an aminoglycoside antibiotic gentamicin sponge. The chest wall was closed by simply suturing the soft tissue.

All patients were intravenously treated with antibiotics; if necessary, treatment was adapted and occasionally modified according to antibiogram results. When the pleural cavity was macroscopically peaceful and the patients had two microbiological negative cultures or when the microbiological culture did not show any further pathogenic bacteria colonisation, antibiotics were discontinued after 7 days.

In the case of BPF, the stump was closed by circumferential suturing of an intra- or extrathoracic muscle flap or pericardial flap. In patients with post(bi)lobectomy empyema in association with necrotizing pneumonia, surgical management consisted of completion pneumectomy (*n* = 3) and muscle flap or pericardial flap transposition followed by intrathoracic VAC therapy. In the absence of BPF, debridement and local decortication were conducted before negative pressure wound therapy. To avoid direct contact of the VAC device with the mediastinum after lobectomy and pneumectomy, the area was covered with a gentle wound contact layer with open mesh design which enables good transfer of exudate to a secondary dressing and easy delivery of topical treatments (Mepitel®, Mölnlycke Health Care, Erkrath-Unterfeldhaus, Germany), during the first session.

**Fig. 2:**
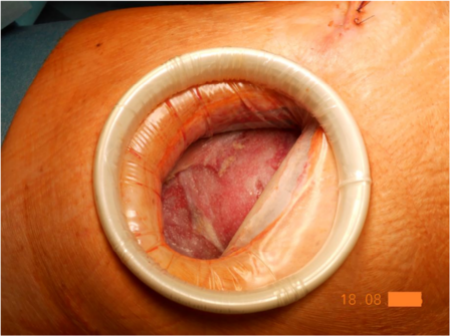
Good macroscopic cover of granulation tissue of visceral pleura

Chest wall closure required two sterile cultures and complete macroscopic cover of granulation tissue of mediastinal and visceral pleura. Furthermore, the presence of viable tissue due to manipulation was eligible (Fig. 2.).

### Statistical analysis

Continuous variables are presented as mean ± standard deviation (SD) or range, categorical variables as absolute numbers and relative frequencies. Categorical variables were compared with chi-square tests. Differences in the duration of intrathoracic vacuum therapy and length of hospital stay among the different surgical techniques were analyzed using analysis of variance (ANOVA). Estimated marginal means (EMM) of the duration of vacuum therapy and the length of hospital stay − adjusted by the type of surgical indication (primary vs. postoperative empyema) − were computed and compared among the different surgical techniques. Paired differences and the corresponding 95 % confidence intervals are presented as effect estimates.

Overall survival time was defined as the date of commencing OWT-VAC or Mini-VAC (Instill) treatment to the date of death or the last follow-up. Univariate Cox proportional hazard models were used to analyze the relationship between overall survival and the variables of age, malignancy, surgery indication, empyema treatment, and successful chest wall closure. Hazard ratios and corresponding 95 % CIs are presented as effect estimates. A *p*-value of < 0.05 was considered statistically significant. Due to the exploratory nature of this study, no adjustments for multiple testing were made. All statistical analyses were done with the IBM SPSS software program Version 23.0 (IBM Corporation, Armonk, New York, United States).

## Results

### Short-term outcomes

Overall, the mean duration of intrathoracic vacuum therapy was 14 days, ranging from 5 to 48 days. Paired comparison of the VAC procedures (Table 2) showed that the mean vacuum duration in the Mini-VAC and Mini-VAC-Instill groups (12.4 ± 5.7 and 10.4 ± 5.4 days) was significantly shorter than that in the OWT-VAC group (20.3 ± 9.4 days). The mean durations of VAC in the primary empyema group and postoperative empyema group were almost equal (14.4 days vs. 14.3 days; *p* = 0.968).

**Table 2:** ANOVA post-hoc pairwise comparisons of OWT-VAC, Mini-VAC, and Mini-VAC-Instill regarding vacuum duration and length of hospital stay

	Vacuum duration in days^a^		
Pairwise therapy comparisons	Estimated marginal means	Difference (95 %-CI)	*p*-value
OWT-VAC vs Mini-VAC	20.3 vs 12.4	7.9 (1.0; 14.8)	**0.026**
OWT-VAC vs Mini-VAC-Instill	20.3 vs 10.4	9.9 (4.27; 15.5)	**0.001**
Mini-VAC vs Mini-VAC-Instill	12.4 vs 10.4	2.0 (−4.9; 8.8)	0.565
	Postoperative length of stay in hospital in days^b^		
Pairwise therapy comparisons	Estimated marginal means	Difference (95 %-CI)	*p*-value
OWT-VAC vs Mini-VAC	24.4 vs 23.7	0.7 (−8.7; 10.1)	0.881
OWT-VAC vs Mini-VAC-Instill	24.4 vs 15.6	8.8 (1.1; 16.6)	**0.027**
Mini-VAC vs Mini-VAC-Instill	23.7 vs 15.6	8.2 (−0.8; 17.1)	0.074

*95 %-CI* 95 % confidence interval

^a^Main effects: Vacuum duration (*p* = 0.003), surgery indication (*p* = 0.968); ^b^Main effects: Vacuum duration (*p* = 0.052), surgery indication (*p* = 0.744)

No major complication was related to intrathoracic VAC therapy. The median number of VAC changes was 2 (range: 1 to 6 changes). In the OWT-VAC group, the chest wall was closed secondarily after recovery in 12 (60 %) of 20 patients (Table 3). In the minimally invasive vacuum therapy groups, all patients but one underwent primary chest wall closure during the same hospital stay (22 of 23, 96 %).

**Table 3:** Pairwise comparison of different OP procedures for primary chest wall closure

OP Technique	Chest wall closure	Paired comparison of success
OWT-VAC *n* = 20	12/20 (60 %)	OWT-VAC vs. Mini-VAC, *p* = 0.034
Mini-VAC *n* = 8	8/8 (100 %)	Mini-VAC vs. Mini-VAC-Instill, *p* = 0.455
Mini-VAC-Instill *n* = 15	14/15 (87 %)	Mini-VAC-Instill vs. OWT-VAC, *p* = 0.026

The success rate was higher (*p* = 0.034, Table 3.) in the minimally invasive vacuum group (8 of 8, 100 %) than in the OWT-VAC group (12 of 20, 60.0 %). The success rate was also higher (*p* = 0.026) in the minimally invasive vacuum group with instillation (14 of 15, 93.3 %) than in the OWT-VAC group (12 of 20, 60.0 %). The success rates in the two minimally invasive groups were similar (*p* = 0.455).

The mean postoperative length of stay (LOS) in hospital of all patients was 21 days (median 18, range 6 to 51 days, Table 2). LOS was significantly shorter (*p* = 0.027) in the Mini-VAC-Instill group (15.1 ± 4.8) than in the other two groups (23.8 ± 12.3, 22.7 ± 1.5). The mean LOS did not differ between the primary empyema group and the postoperative empyema group (21.8 days vs. 20.6 days; *p* = 0.744).

Overall, the 30-day and 60-day mortality rates were 4.6 % (2 of 43) and 9.3 % (4 of 43), and none of the deaths was related to infection.

### Long-term outcomes

In 9 patients, fenestrations were left open; 2 patients died before closure, 3 rejected reoperation, 4 had permanent bacterial colonization of the window, and 1 was treated with definitive OWT because of acute recurrence after primary closure. Therefore, the overall success rate for the final closure of the chest wall without empyema recurrence was 76.7 % (33 of 43).

**Fig. 3:**
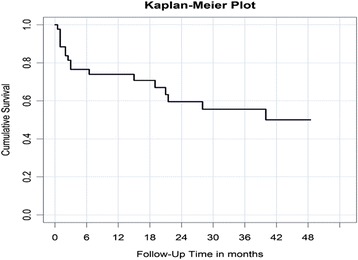
Cumulative survival

The 1-year, 2-year, 3-year, and 4-year survival rates were 74.6, 60, 56, and 50 % respectively (Fig. 3). There were no significant differences in the 4-year survival rates of the three subgroups (40 % in the OWT-VAC, 62.5 % in the Mini-VAC, and 60 % in the Mini-VAC-Instill group).

No case of death was related to intrapleural vacuum therapy. The Cox regression analysis of overall survival showed no prognostic factors (Table 4). Only malignancy showed a slight tendency (HR = 2.37 (0.87-6.41), *p* = 0.091).

**Table 4:** Relationship between investigated factors and survival

Factors	Survival	
	HR (95 %-CI)	*p*-value
Age	1.03 (0.99–1.08)	0.152
Malignancy (yes/no)	2.37 (0.87–6.41)	0.091
Surgery indication:		
Primary empyema	Reference	-
Postoperative empyema	0.60 (0.22–1.61)	0.306
Empyema treatment:		
OWT-VAC	Reference	-
Mini-VAC	0.66 (0.18–2.35)	0.518
Mini-VAC-Instill	0.61 (0.16–2.29)	0.460
Successful final chest wall closure (yes/no)	0.81 (0.29–2.31)	0.695

## Discussion

Treatment of debilitated and pre-septic or septic patients with pleural empyema is still a challenge. Even in the case of successful empyema management, patients with multimorbidity, malignancy, and/or immunosuppression have a worse short-term prognosis (mortality up to 15 %, Table 5) and long-term prognosis with a median survival of 22.8 to 67 months [7, 8] than patients without such additional complications.

**Table 5:** Overview; outcomes of OWT therapy in debilitated patients

Author/Year	Number of patients	Empyema cause	30-day mortality	Closure of OWT	Recurrence	OWT duration
Maruyama et al. 2001	53	Heterogeneous	15 % (8/53)	83 % (44/53)	11 % (5/44)	Mean 128.0+/−32.1 days
Thourani et al. 2003	78	Heterogeneous	5 % (4/78)	definitive OWT	definitive OWT	definitive OWT
Massera et al. 2009	19	Heterogeneous	6 % (1/17)	59 % (10/17)	10 % (1/10)	Median 5 (3-9 months)
Reyes et al. 2010	78	Heterogeneous	6 % (5/78)	22 % (17/78)	6 % (1/17)	Median 454 (90–1068 days)

Our study showed that intrathoracic VAC technique offers a safe treatment option for pleural empyema with good short-term and long-term outcomes, particularly in debilitated patients.

### Short-term outcome

Minimally invasive treatment with Mini-VAC and Mini-VAC-Instill significantly reduces the duration of vacuum treatment in comparison to OWT-VAC. The length of stay in hospital could also be reduced by the Mini-VAC(−Instill) procedure, although − in almost all minimally invasive treated patients (22/23) − final chest wall closure was conducted during the same hospital stay, which additionally extended LOS. In addition, a previous study of our group showed the efficacy of wound flushing in combination with vacuum in invasive aggressive empyema thoracis – i.e. highly aggressive bacteria or reduced immunity of patients [9].

So, the last treatment period (2013 to 2014) showed that both treatment duration and LOS could be significantly reduced by Mini-VAC-Instill therapy in comparison to the OWT-VAC and Mini-VAC technique.

To date, only few authors have achieved similar LOS for thoracic empyema [10, 11]. Schneiter at al. reported a mean hospitalization of 18 days for early and late postpneumectomy empyema with repeated open surgical debridements and antimicrobial therapy [10]. The thorax was definitively closed in 71 of 75 (94.6 %) patients. Thourani et al. treated 78 empyema patients with definitive OWT (modified Eloesser flap) and reported a mean LOS of 16 days [11]. But the modified Eloesser flap procedure was intended as a permanent one-stage procedure. So, in the study by Thourani et al., treatment duration was equivalent to LOS.

In our opinion, the dressings should be routinely changed every three to four days, in the operating room, to allow precise and continued monitoring of infection. In the presence of residual lung tissue we often use the thoracoscope for better visualisation of poorly visible areas.

This means that repeat debridement treatment can be performed as required to keep the wound bed clean for optimal healing.

We treated two patients with definitve BPF and in all the installation of vacuum was possible. In one patient with a one mm fistula, the BPF was sufficiently closed after VAC therapy. The other BPF, with a diameter of eight millimetres, could not be closed by VAC, which was not a problem in the VAC treatment. Future studies should investigate the diameter of BPF that can be closed by negative pressure in VAC therapy.

The largest literature series of OWT patients with heterogeneous causes of empyema (excluding postpneumectomy empyema) showed 30-day mortality rates between 5 and 15 % (Table 5). The OWT procedure may result in a good prognosis in medical unstable and debilitated patients. The most common causes of death after OWT are sepsis and multiorgan failure [7, 8, 11–13]. In our study, we lost only 2 patients due to cardiac and multiorgan failure, which resulted in a 30-day mortality rate of 4.6 % (2 of 43). Therefore, VAC treatment, particularly of the minimally invasive type, offers the same survival prognosis than classical OWT treatment but a higher rate of chest wall closures.

The most important advantage of Mini-VAC and Mini-VAC-Instill therapy is probably immediate chest wall closure after VAC treatment. We preferred conducting chest wall closure during the same hospital stay to avoid later complications including hospitalization and to improve quality of life. In the Mini-VAC group, all thoracic windows were closed, and only one definitive fenestration was left in the Mini-VAC-Instill group. Indispensable requirements for chest wall closure were good macroscopic aspects and negative microbiological cultures. In the study by Palmen et al. [14], 50 % of patients died of OWT-related complications (bleeding and recurrent infections) during follow-up.

Although empyema thoracis recurred once in the Mini-VAC-Instill group, there were no pitfalls on this case.

A recent Cox proportional hazard model showed a significant association of the closure of OWTs (HR 0.31, 95 % CI 0.10–0.88; *p* = 0.03) with overall survival [7]. In our study, successful chest wall closure was not significantly associated with survival (HR 0.81, 95 % CI 0.29–2.31; *p* = 0.695).

### Long-term outcome

The 4-year survival rate of all patients was 50 %, and no case of death was related to intrapleural vacuum therapy. We found no prognostic factor for death or survival, and even malignancy had a *p*-value of 0.091 (Table 4).

Reyes et al. achieved survival rates of 74 and 60 % at 12 months and 60 months [13], when treating 78 debilitated patients with empyema with OWT. In the study by Hato et al., the 60-month survival rate of OWT patients was 34.7 % (12 of 35), whereas the median survival period was 22.8 months. According to the univariate analyses, variables significantly associated with increased overall survival included closure of the OWT (*p* = 0.03) and absence of diabetes mellitus (*p* = 0.04) [7]. The long-term survival of this vulnerable patient population is mainly determined by secondary diseases rather than by the type of treatment. Taking this fact into consideration, the primary goal should be prompt empyema therapy to enable treatment of the underlying disease.

No patient developed systemic symptoms due to the instillation of the 0.02 % polyhexanide solution. We have not seen a negative effect of VAC-(Instill) on the pleura carcinosis. Nevertheless, the use of VAC-Instill therapy can trigger clinically significant complications and there are also some contraindications [9], including: coagulopathy, larger bronchial stump insufficiency, ongoing pain and contact allergy or anaphylactic reactions. Because of this, cautious monitoring of the VAC system must be exercised in the hospital. Therefore, outpatient management for Mini-VAC-Instill is not recommended.

Although the cost of materials in the Mini VAC (−Instill) groups was considerably higher than in the OWT-VAC group, in the final effect, on grounds of the shorter LOS, the treatment costs were lower in the minimally invasive groups.

### Study limitation

The main limitations of our study are its design as a retrospective single-center study and the limited number of patients included. To overcome these biases, a prospective multicenter study should be conducted.

## Conclusions

Minimally invasive VAC treatment (Mini-VAC and Mini-VAC-Instill) fulfills the aim of a safe and fast therapy in debilitated and near-septic or septic patients with empyema. The great advantage of minimally invasive VAC treatment is the high rate of chest wall closure during the same hospital stay. This offers better quality of life for the patients, a very low reinfection rate, and fast treatment of secondary diseases (e.g. tumour therapy). Additional instillation therapy is particularly beneficial for septic patients with highly infected or bacterially colonized pleural empyema. Based on our data, we recommend generally the use of Mini-VAC-Instill in the abscence of BPF. Mini-VAC is also one of the choices if there is a permanent supply of causative organisms due to a smaller than 1 cm BPF. If there is a large BPF (≥ 1 cm), we recommend muscle or pericardium flap transposition after OWT-VAC pretreatment. The techniques presented support a non-delayed application of intrathoracic vacuum-closure therapy.

## Abbreviations

ANOVA: Analyzed using analysis of variance
ATS: American Thoracic Society
BPF: Bronchopleural fistula
CT-guided: Computertomography guided
EMM: Estimated marginal means
LOS: Length of stay in hospital
Mini-VAC: Minimally invasive vacuum-assisted closure
Mini-VAC-Instill: Minimally invasive vacuum-assisted closure with instillation
OWT-VAC: Open window thoracostomy with vacuum-assisted closure
VAC: Vacuum-assisted closure
